# Discordant renal progression of Fabry disease in male monozygotic twins: a case report

**DOI:** 10.3389/fgene.2023.1150822

**Published:** 2023-06-14

**Authors:** Do-Yun Lee, Jun-Yeong Jeong, Seung-Eun Lee, Jae-Hun Lee, Ju-Young Moon, Su Woong Jung, Sang-Ho Lee, Yang Gyun Kim

**Affiliations:** ^1^ College of Medicine, Kyung Hee University, Seoul, Republic of Korea; ^2^Division of Nephrology, Department of Internal Medicine, College of Medicine, Kyung Hee University, Seoul, Republic of Korea

**Keywords:** Fabry disease, male monozygotic twins, genotype-phenotype discordance, discordant renal progression, epigenetics

## Abstract

**Background:** Fabry disease (FD) is a rare X-linked lysosomal storage disease caused by mutations in the *GLA* gene that encodes α-galactosidase A (α-GAL). Clinical phenotypes tend to vary in monozygotic female twins because mutations are located on the X-chromosome, whereas similar phenotypes are found in male monozygotic twins. Here we report the case of male monozygotic twins with FD presenting with distinguishable renal phenotypes.

**Case:** A 49-year-old male patient who visited the hospital with proteinuria 14 years prior was readmitted for the same issue. His monozygotic twin brother had started hemodialysis 6 months prior due to renal failure of unknown origin. The patient’s renal function was within the normal range, while his spot urine protein-to-creatinine ratio was 557 mg/g. Echocardiography revealed left ventricular hypertrophy (LVH). The findings of a renal biopsy were consistent with FD. Genetic testing identified a c.656T>C mutation in the *GLA* gene, and α-GAL activity was significantly decreased. Genetic screening of his family clarified that his mother, older sister, twin brother, and his daughter had the same genetic mutations. The patient received enzyme replacement therapy 34 times. Subsequently, migalastat was initiated that continues today. Renal function and proteinuria remain stable, and the LVH has mildly improved.

**Conclusion:** This is the first case of male monozygotic twins expressing different progressions of FD. Our findings demonstrate the possibility that environmental or epigenetic factors may critically influence genotype–phenotype discordance.

## 1 Introduction

Fabry disease (FD) is a rare X-linked recessive lysosomal storage disease caused by the lack of the hydrolytic enzyme, α-galactosidase A (α-GAL), which is necessary for glycosphingolipid metabolism in lysosomes (Mahmud, 2014; Bernardes et al., 2020). More than 900 different mutations of the *GLA* gene responsible for FD have been identified (Bernardes et al., 2020). FD can be classified into two subtypes: In classic FD, with less than 1% α-GAL activity, multiple clinical manifestations, such as limb pain, abnormal sensation, angiokeratoma, cardiomyopathy, gastrointestinal discomfort, and sweating reduction, appear in childhood or adolescence (Michaud et al., 2020); in late-onset FD, with 2%–30% α-GAL activity, particular organ involvement is dominant without skin or nervous symptoms, and disease onset is relatively late (Desnick and Brady, 2004; Mahmud, 2014; Bernardes et al., 2020). The cardiac variant is the most common, followed by the renal form of FD (Kubo, 2017). Slowly progressive FD induces problems in the kidneys, heart, and nervous system, mostly at age 40–60 years (Chan and Adam, 2018). Due to the disease characteristics of X-linked mutations, female patients with FD tend to have various levels of α-GAL activity and milder phenotypes than their male counterparts. Several case studies have demonstrated that phenotypes can differ in females, even in monozygotic twins (Marguery et al., 1993; Redonnet-Vernhet et al., 1996). In contrast, monozygotic male twins with FD have tended to show identical phenotypes (Gomez et al., 2012). Here we describe our encounter with a pair of monozygotic male twins with completely different renal manifestations. One brother started hemodialysis due to FD-induced renal failure 6 months prior, while the other twin had normal renal function with mild proteinuria. Our findings suggest that genetic mutations as well as epigenetic influences may be critical for the progression of FD-associated organ damage.

## 2 Case report

**FIGURE 1:**
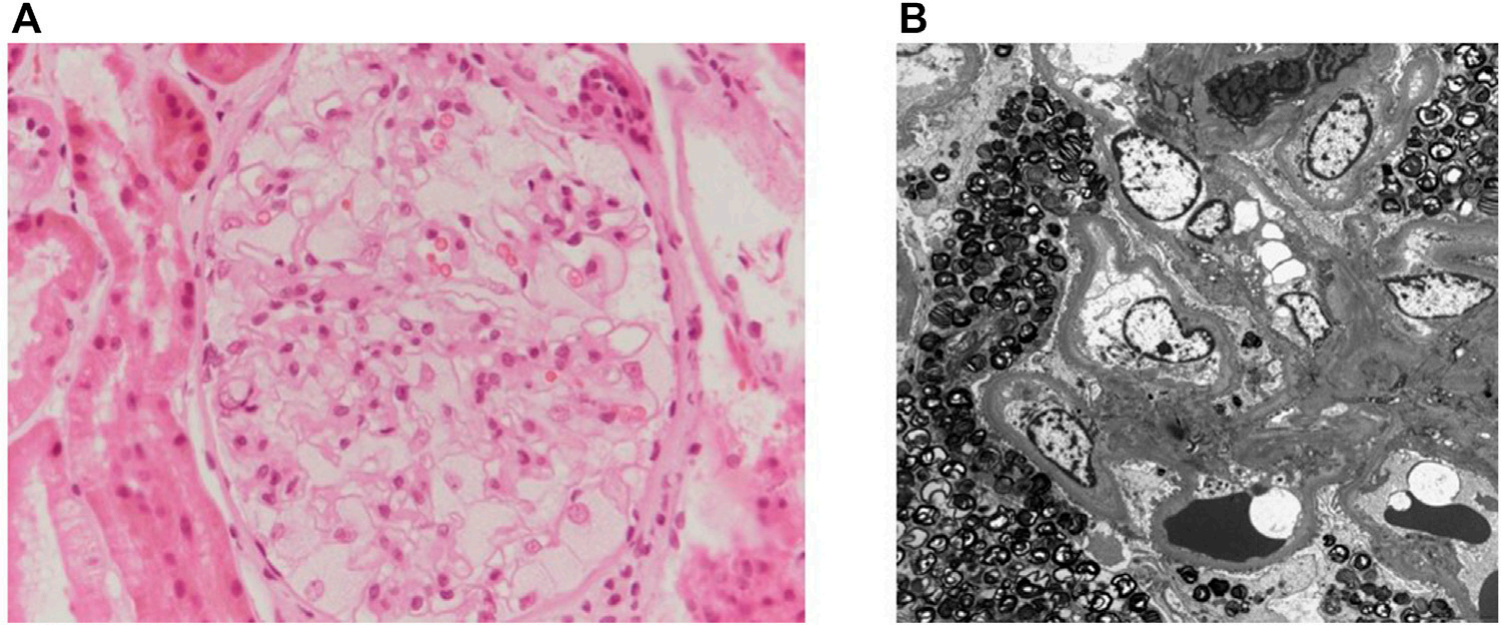
The patient’s renal biopsy. **(A)** Renal pathology under light microscopy with periodic acid-Schiff stain (×400). Foamy cytoplasm is seen in the capillary lumen of the glomeruli. **(B)** Electron microgram showing laminated lipid droplets and diffuse foot process effacement in the glomerular epithelium.

A 49-year-old man was admitted to our hospital for proteinuria in April 2020. The patient was diagnosed with hypertension and mild proteinuria in 2006. Irbesartan (75 mg/day) was prescribed, but the patient had not taken it regularly. His left ear became deaf 40 years prior for no particular reason. The patient was a monozygotic twin. His twin brother had started hemodialysis 6 months prior due to renal failure of unknown cause. The patient was planning to donate one of his kidneys to his twin brother. The blood chemistry results were as follows: blood urea nitrogen, 12 mg/dL (normal range: 8–20 mg/dL); creatinine (Cr), 0.85 mg/dL (normal range: 0.67–1.17 mg/dL); and estimated glomerular filtration rate by Modification of Diet in Renal Disease equation, 95.8 mL/min/1.73 m^2^ (normal range >60 mL/min/1.73 m^2^). The spot urine protein/Cr ratio was 557.2 mg/g (normal range <200 mg/g), while the 24-h urine protein and albumin levels were 815.4 mg (normal range <150 mg/day) and 591.2 mg (normal range <30 mg/day), respectively. Urine red blood cells were counted at two to four per high power field (normal range: 0–1/HPF). Blood electrolytes (Na 140/K 4.5/Cl 103 mEq/L, normal range: Na 134–144, K 3.5–5.1, Cl 101–109 mEq/L) and the total CO_2_ level (28.9 mmol/L, normal range: 21–31 mEq/L) were within the normal ranges. Autoantibodies, including antinuclear antibodies, anti-neutrophil cytoplasmic antibodies, anti-glomerular basement membrane antibodies, and anti-phospholipase A2 receptor immunoglobulin G, were negative. Complement 3 was 125.0 mg/dL, complement 4 level was 35 mg/dL, which were within the normal ranges (normal range: C3 90–180, C4 10–40 mg/dL). There were no monoclonal peaks in the serum or urine electrophoresis. A renal biopsy performed to clarify the cause of the proteinuria. Foamy cytoplasms were found in the capillary lumen of the glomeruli under the light microscopy (Figures 1A). Electron microscopic images identified laminated lipid droplets and diffuse foot process effacement in the glomerular epithelium, features consistent with FD (Figures 1B).

**FIGURE 2:**
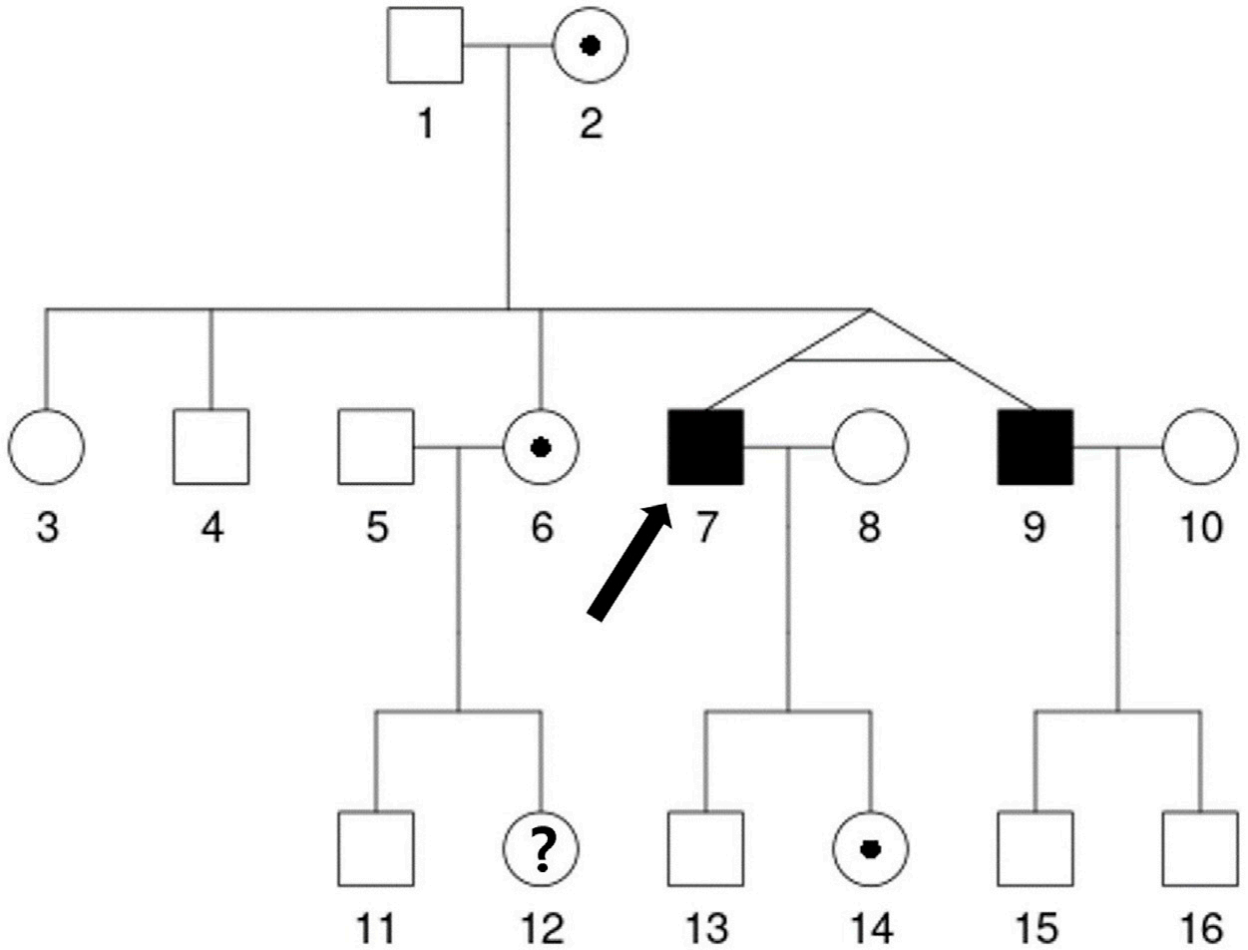
The patient’s family pedigree. The genetic analysis showed that the proband (7, denoted with an arrow) has the c.656T>C mutation in *GLA* gene exon 5, which is likely pathogenic for Fabry disease. After his diagnosis, his mother (2), older brother (4), older sisters (3, 6), monozygotic twin brother (9), daughter (14), and older sister's son (11) underwent genetic analyses. His older sister’s daughter (12) declined to undergo the genetic analysis. The same mutation was found in his monozygotic twin brother (9), one of his older sisters (6), his daughter (14) and his mother (2). The age of some relatives is as follows: Proband (7) and his monozygotic twin brother (9) 52 years, sister (6) 54 years, brother (4) 56 years, father (1) 82 years and mother (2) 81 years.

Analyses of α-GAL activity and genetics were also performed. The patient’s plasma α-GAL activity was 5.13% of mean normal level (0.30 μmol/h/L), while the plasma globotriaosylsphingosine (lyso-Gb3) level was 10.30 ng/mL (normal range <1.74 ng/mL). He had a c.656T>C (p.Ile219Thr) mutation in *GLA* gene exon 5 that was classified as a likely pathogenic mutation of FD according to the 2015 American College of Medical Genetics/Association for Molecular Pathology guidelines (Richards et al., 2015). Chest radiography and electrocardiography revealed cardiomegaly and a first-degree atrioventricular block with a normal heart rate. Echocardiography findings showed left ventricular hypertrophy (LVH) with a left ventricular mass index of 164.7 g/m^2^ (normal range <115 g/m^2^). Three autonomic tests were conducted. Sympathetic skin response evaluates sudomotor (sympathetic) function, and heart rate response to deep breathing and 30:15 ratio examines cardiovagal (parasympathetic) function. (Cheshire et al., 2021). The patient showed poor sympathetic skin response on both feet, and abnormal heart rate response and 30:15 ratio, which suggested dysfunction of both sympathetic and parasympathetic function. Brain magnetic imaging showed basilar arterial dolichoectasia being suggested the Fabry disease-associated changes. An ophthalmological examination revealed no abnormalities. The patient’s family members were tested for FD, which confirmed that his mother, older sister, twin brother, and daughter harbored the same *GLA* mutation (Figure 2). The short tandem repeat analysis showed 100% agreement between his twin brother and him, which demonstrated they were monozygotic twins. His twin brother started enzyme replacement therapy (ERT) to prevent FD progression in cardiovascular problems. The α-GAL activity of his twin brother was 6.15% of mean normal level (0.36 μmol/h/L) before the ERT initiation. His older sister and daughter had normal α-GAL activity without clinical manifestations; thus, periodic follow-ups at our clinic have been planned. Recently, his mother complained of peripheral pain, so ERT was considered.

For our patient, ERT was started immediately after diagnosis and performed 34 times. His gene mutation was amenable to use the pharmacologic chaperon ‘migalastat’. ERT was replaced with migalastat because the patient wanted to save time and avoid injections. The patient has since been continuously treated with migalastat. A follow-up lyso-Gb3 test was conducted every 6 months. The plasma lyso-Gb3 level decreased from 10.30 ng/mL to 4.38 ng/mL, while the α-GAL activity increased from 5.13% (0.30 μmol/h/L) to 23.25% (1.36 μmol/h/L) of mean normal level at 3 months after ERT. After changing over to migalastat, his lyso-Gb3 levels remained well-controlled at 2–4 ng/mL. The patient’s renal function was stable, and no significant change in the proteinuria was noted. Echocardiography conducted after 1 year of migalastat treatment demonstrated a slightly decreased left atrial size and regressed LVH with a left ventricular mass index of 144.1 g/m^2^.

## 3 Discussion

Here we described our encounter with a patient with mild proteinuria due to FD with a monozygotic twin brother who started hemodialysis due to FD-induced end-stage renal disease. Male monozygotic twins generally have similar phenotypes since they share the same mutation on a single gene on the X chromosome (Zwijnenburg et al., 2010). In contrast, clinical manifestations may differ in female monozygotic twins with FD because of X-inactivation (Marguery et al., 1993; Redonnet-Vernhet et al., 1996), a process in which one copy of the X-chromosome can be transcriptionally silenced in female cells (Loda et al., 2022). An imbalance of maternal and paternal X-chromosomal expression is common, so X-inactivation may affect the phenotype of female carriers of X-linked diseases (Shvetsova et al., 2019). Since X-inactivation does not occur in men, male monozygotic twins with X-linked disorders usually show similar phenotypes (Gomez et al., 2012). This is the first reported case of monozygotic twins with FD showing different renal stages.

Environmental factors, such as diet and smoking, can influence the phenotypic differences between monozygotic twins (Wong et al., 2005). In autosomal dominant polycystic kidney disease, environmental factors, including caffeine intake, smoking, and obesity, can influence renal manifestations (Rossetti and Harris, 2007). In a previous report, three adult men of one family with the same FD mutations showed different disease phenotypes, especially renal manifestations (Mignani et al., 2017). The authors suggested that environmental factors and modifier genes could affect phenotypes. However, in monozygotic twins, epigenetics may have a greater impact than environmental factors (Petronis, 2010). Epigenetic differences, such as DNA methylation and histone modification, have been reported in one of three monozygotic triplets (Fraga et al., 2005). The intrauterine environment induces different epigenetic changes in monozygotic twins (Ollikainen et al., 2010).

In X-linked diseases, such as Wiskott-Aldrich syndrome and Kennedy disease, monozygotic male twins with different phenotypes have been reported and epigenetic changes such as DNA methylation were the purported reason (Buchbinder et al., 2011; Popescu, 2019). The loose genotype–phenotype connection in several FD cases may be caused by different epigenetic regulations (Hassan et al., 2017). The c.656 T>C (p.I219T) mutation, found in our patient’s genetic test, is considered a late-onset variant (Hwu et al., 2009). In another report, a 4-year-old boy with a *GLA* gene mutation, known as the late-onset variant, showed the classic FD clinical phenotype (Hirashio et al., 2021). Considering the patient’s age, epigenetic factors may impact the FD phenotype more significantly than environmental factors. Interestingly, our patient and his brother lived in a similar environment, and they reported that their lifestyles, including smoking and drinking habits, did not differ. As such, epigenetic factors probably had a more significant impact on the progression of FD in this case.

Our patient was started on ERT to inhibit renal and cardiac deterioration. However, since it is administered intravenously, it requires a hospital visit every 2 weeks for more than 2 h each time. Thus, lifelong ERT maintenance can significantly disrupt one’s daily life. In contrast, migalastat is a pharmacological chaperone that increases α-GAL activity and is amenable to limited variations (Germain et al., 2016). Korean insurance allows patients with FD to change from ERT to migalastat after at least 1 year of ERT. The patient had an amenable variation (c.656 T>C, p.Ile219Thr) for migalastat; thus, he was started on migalastat after 17 months of ERT treatment. After changing therapy, our patient’s renal function remained stable; thus, the migalastat was continued until now. Lyso-Gb3 4–5 ng/mL was decreased to 2.49 ng/mL at 6 months after the initiation of migalastat. As the patient’s monozygotic twin brother showed cardiac symptoms, he was also started and maintained on ERT.

This is the first reported case of male monozygotic twins with different stages of renal disease in FD. Our findings suggest the possibility that epigenetic factors may critically influence genotype–phenotype discordance.

## Data Availability

The raw data supporting the conclusion of this article will be made available by the authors, without undue reservation.
